# Exploration of Circulating Tumour Cell (CTC) Biology: A Paradigm Shift in Liquid Biopsy

**DOI:** 10.1007/s12291-020-00923-4

**Published:** 2020-09-16

**Authors:** Anshika Chauhan, Rajandeep Kaur, Sushmita Ghoshal, Arnab Pal

**Affiliations:** 1 Department of Biochemistry, Post Graduate Institute of Medical Education and Research, Chandigarh, India; 2 Department of Radiotherapy, Post Graduate Institute of Medical Education and Research, Chandigarh, India

**Keywords:** Liquid biopsy, Circulating Tumour Cells (CTCs), Diagnosis, Prognosis, Molecular characteristics of CTCs, Genomics, Transcriptomics, Proteomics, Cancer staging

## Abstract

Circulating tumour cells (CTCs), are disseminated tumour cells found in the blood in solid tumour malignancies. Identification of CTCs act as emerging tools in the field of the Liquid Biopsy. Majority of the studies focused on detection and enumeration of CTCs due to technological challenges those results from the rarity of CTCs in the blood. Enumeration of CTCs has already proven their value as prognostic as well as predictive biomarkers for disease prognosis. However, recent advances in technology permitted to study the molecular and functional features of CTCs and these features have the potential to change the diagnostic, prognostic and predictive landscape in oncology. In this review, we summarize the paradigm shift in the field of liquid biopsy-based cancer diagnostics using CTC isolation and detection. We have discussed recent advances in the technologies for molecular characterization of CTCs which have aided a shift from CTC enumeration to an in-depth analysis of the CTC genome, transcriptomes, proteins, epigenomes along with various functional features. Finally, as a prognosticating strategy, the potentials of CTCs as a tool of liquid biopsy to predict micrometastasis, monitor prognosis and how to use them as an additional tool for cancer staging has been discussed.

## Introduction

Circulating Tumour Cells (CTCs) are the cancer cells those detach from a solid tumour lesion and enter the circulation. CTCs contain a specific population of precursors cells that are responsible for the metastatic process and there is a great deal of interest in using them as a tool of liquid biopsy to diagnose the micro-metastatic disease as well as to monitor and predict the course of the disease. The isolation of viable CTCs in the unaltered or minimally changed state was limited for a long time due to technological challenges owing to the rarity of CTCs in comparison to normal blood cells. However, with the recent advances in high throughput technologies for analyzing single-cell biology made during the last decade, interrogation of CTCs at the molecular and functional level and to assess their suitability for clinical applications have gained interest. Enumeration of CTCs has already proven their value as prognostic as well as predictive biomarkers for disease prognosis. Also, their suitability for additional applications is being tested. In this review, we summarize available methods for CTC isolation and detection which have great potential to be used in the field of liquid biopsy. We will also discuss recent advances in the technologies for molecular characterization of CTCs which have enabled a transition from CTC enumeration to a detailed analysis of their genome, transcriptomes, proteomes, epigenomes and various functional features. Finally, we will discuss the potential of CTCs as a tool of liquid biopsy to predict micro-metastasis, monitor prognosis and to use them as an additional tool for cancer staging, along with widely accepted TNM staging.

### Liquid Biopsy and Its Role in Cancer Diagnostics

The term “liquid biopsy” was first used to define various methods that used to derive similar diagnostic information from the blood that is normally derived from a tissue biopsy [1]. While in oncology, this term is used in a comprehensive sense where it refers to the analysis of various biological fluids, mainly blood but also various other easily accessible biological fluids such as saliva. The concept of liquid biopsy focuses on simple, fast and cost-efficient prediction as well as monitoring of disease progression or response to treatment. Liquid biopsy offers numerous advantages over “conventional” tissue biopsy. Liquid biopsy is also less invasive than tissue biopsy as biological fluids like blood, saliva or urine are easily accessible. For instance, it is technically challenging to obtain a successful tissue biopsy in lung cancer. Furthermore, tissue biopsies are not able to appropriately reflect the complex molecular features of a primary tumour, because of temporal or spatial heterogeneity, which required biopsies from different tumour areas. Contrary to this, a more comprehensive cross-section of heterogeneous diseases is provided by liquid biopsies [2].

Various analytes can be analyzed during liquid biopsy include Circulating Tumour Cells (CTCs); Circulating Tumour DNA (ctDNA); circulating cell-free tumour RNA (cfRNA), which contains predominantly small RNAs like miRNAs but also various mRNAs; circulating Extracellular Vesicles (EVs), such as exosomes; Tumour-Educated Platelets (TEPs); various proteins and metabolites [3–8]. While analysed together, these analytes might provide information about various features of primary tumours or metastases. Primarily, these metabolites are used to derive information about genomic aberrations and copy number variations [3] but now liquid biopsies are progressively being used to produce information about the transcriptome [9], the epigenome [10], the proteome [11] and the metabolome [12] of the primary tumour as well as metastases.

### CTCs as a Tool for Liquid Biopsy

**Fig. 1:**
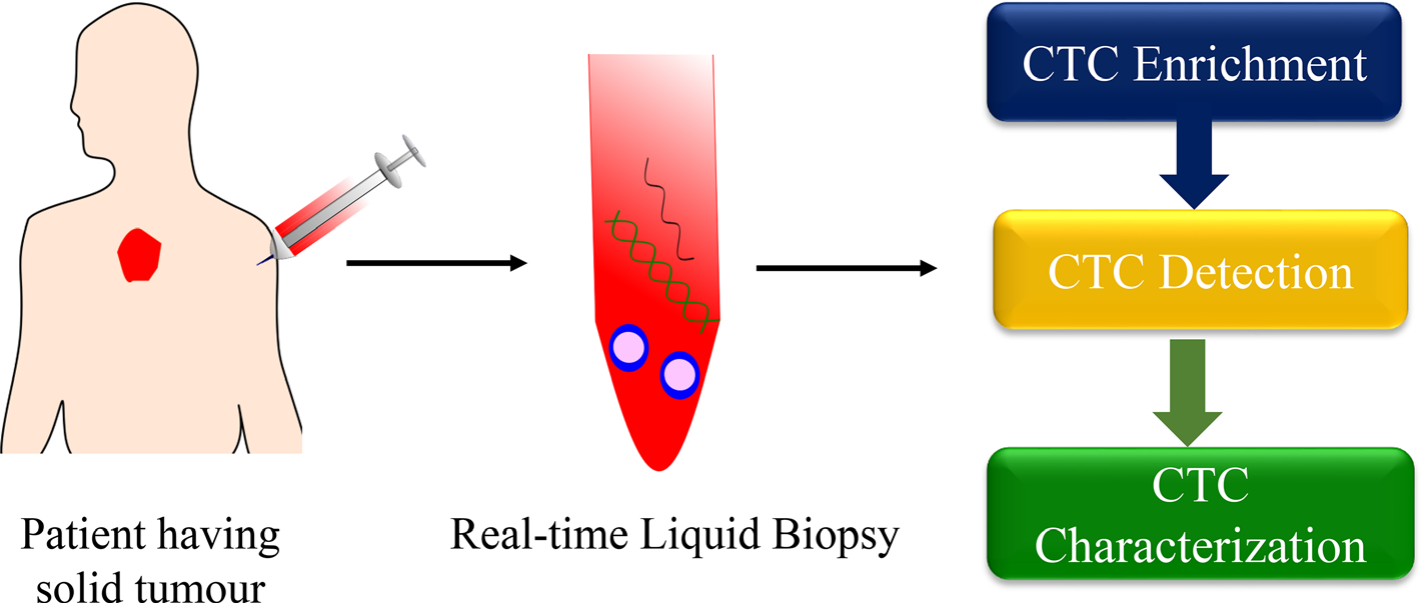
CTCs as a tool for liquid biopsy. CTCs isolated from the blood of patient having solid tumour can be explored as a promising tool for liquid biopsy in real time. CTC enrichment followed by their detection and ultimately characterizing their molecular features could lead to a paradigm shift in the field of liquid biopsy

Circulating Tumour Cells (CTCs) are cells which detach from primary or secondary tumour and enter the bloodstream. Studies have shown that CTCs are responsible for cancer metastases and nowadays, they are explored as a promising component of liquid biopsy. CTCs are known to be circulating in the blood in the primary stages of disease even before clinical evidence of metastases [13]. However, they are hard to be identified, being present in an extremely low proportion concerning the other cells present in the blood. As a result, enrichment of CTCs in the body fluid is an absolute requirement for isolation and identification of these cells (Fig. 1).

#### Enrichment of CTCs

**Fig. 2:**
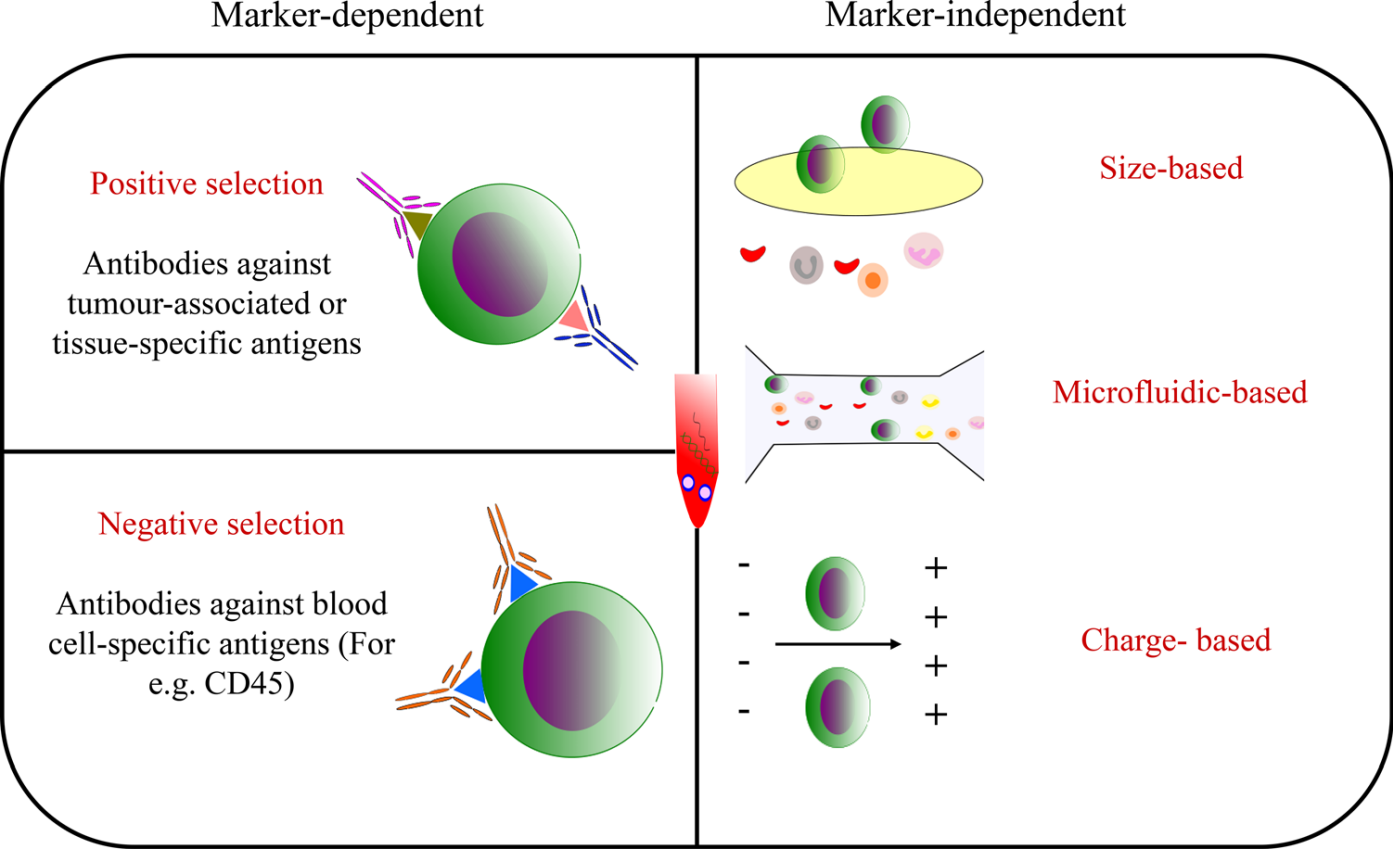
Enrichment of CTCs. Approaches for CTC enrichment either exploit the difference between CTCs and blood cells in their the biological (marker dependent) or physical properties(marker independent). Marker-dependent methods include positive (using antibodies against tumour-associated or tissue-specific antigens) and negative selection (using antibodies against blood cell-specific antigens). Marker independent approach utilizes the differences in physical properties like size, charge etc

Majority of the approaches for CTC enrichment are based upon the differential expression of certain cell surface molecules or the distinctly different physical properties, thus differentiating the tumour and blood cells exploit the differences in the tumour and blood cells that includes the distinct physical and molecular characteristics (Fig. 2). Regarding the marker dependent approach, EpCAM is the most widely used marker for the enrichment of CTCs [14]. However, various other markers such as EGFR [15], prostate-specific membrane antigen (PSMA) [16] in prostate cancer and HER2 in breast cancer-specific CTCs have also been explored. Phenotypically, CTCs, being very heterogeneous do not express the specifically chosen marker in all the cells; therefore, enrichment of CTCs through single positive selection marker generally introduce bias. This problem may be addressed by adding a negative selection strategy. In this strategy, the normal blood cells are depleted to a significant degree, using antibodies specific to various cell-surface antigens expressed on human leukocytes e.g. CD45 and CD34. The major drawback of CTC enrichment through negative selection is that the purity of enriched CTCs is compromised. There is also the risk that the CTCs may be entrapped within the bulk of blood cells, resulting in inadvertent loss of CTCs.

The other approach is the phenotype marker independent. In this strategy, the differences in the physical characteristics such as size, densities, electrical charges and deformability of tumour cells and non-malignant blood cells are exploited. The disadvantage of this approach is that these properties are extremely variable among CTCs with considerable overlap with those of other non-malignant cells. It was reported that the bulk of normal blood cells have a diameter of about 10 μm, whereas the diameter of CTCs is inconsistent, ranges from 6 μm to more than 20 μm [17]. A microfiltration device involves passing the blood through pores with sizes standardized to entrap CTCs, results in size exclusion and ultimately retaining larger CTCs, while it leads to potential loss of small-sized CTCs [18].

#### Detection of CTCs

Even after the enrichment process, the CTC population might also contain a large number of leukocytes, therefore identification of individual CTCs involves the use of various other dependable methods. Immunological identification using antibodies to the various membrane-bound as well as cytoplasmic antigens is the predominant approach for the detection of CTCs. The Cell Search System™ is the first and only clinically validated, FDA approved system for the detection of CTCs. It detects and enumerates CTCs which are of epithelial origin (CD45-, EpCAM+ and Cytokeratins 8, 18+ and/or 19+) in 7.5 mL of peripheral blood [19].

**Fig. 3:**
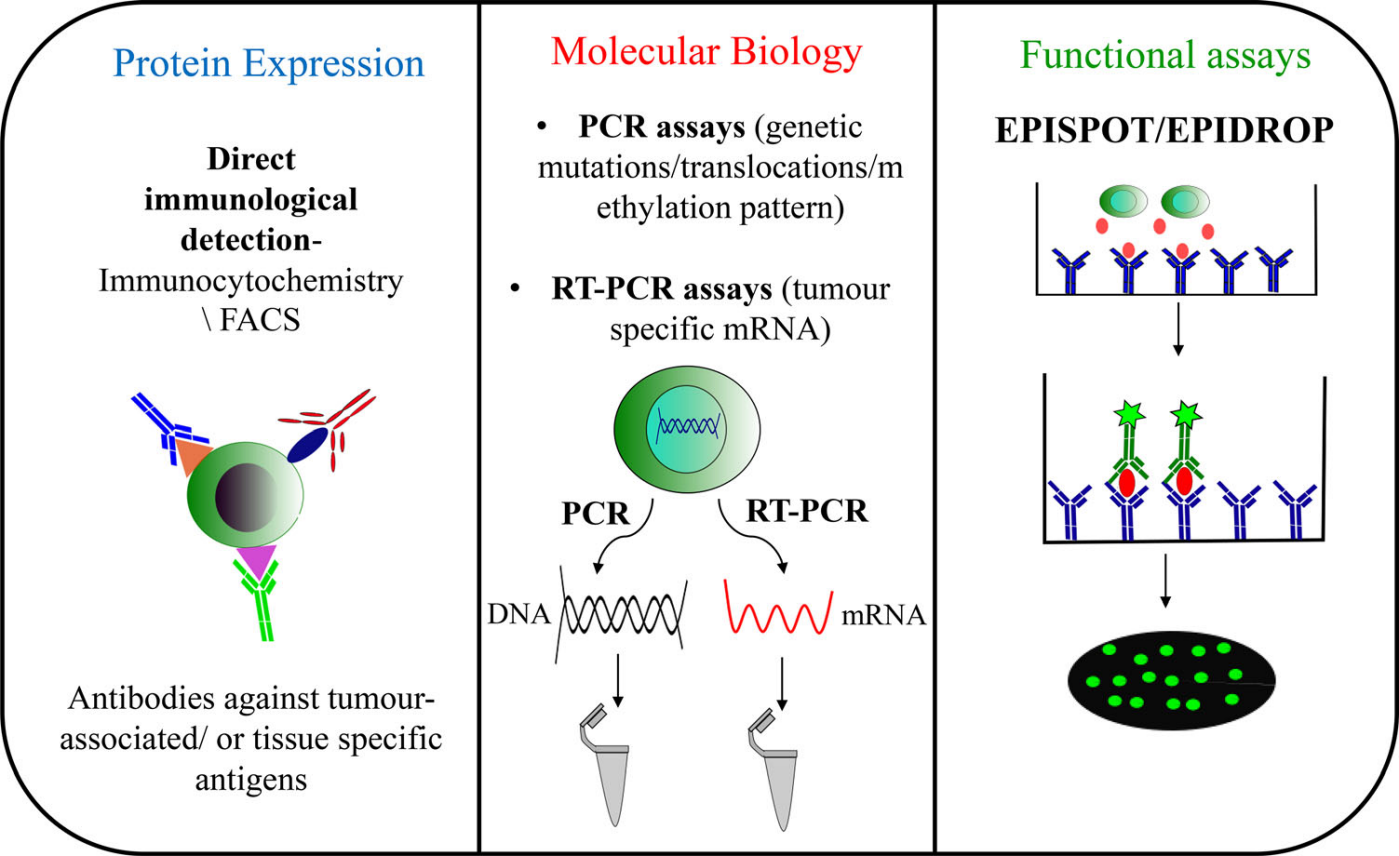
Detection of CTCs. After successful enrichment, CTCs can be detected by using various strategies. Exploiting protein expression pattern of CTCs by using immunocytochemistry/FACS. Molecular biology techniques either include detection of genetic mutations/translocations/methylation pattern using PCR assay or detection of specific mRNAs using RT-PCR. Function properties like specific proteins secreted can also be exploited for CTC detection

An alternative approach which has been used for CTC detection, identification and their phenotypic analysis is fluorescence-activated cell sorting (FACS). However, this approach requires a pre-enrichment step to increase the concentration of CTCs before its detection making it time-consuming and also result in decreased viability of CTCs [20]. This disadvantage of time-consuming pre-enrichment step required for FACS based method was overcome by Lopresti et al. [21]. In this technique, firstly, cells are simultaneously fixed, permeabilized, and stained within a span of 45 min. Secondly, using low-speed acquisition in FACS along with cell size and pan-cytokeratin expression as discriminators, established the protocol for efficient detection of CTCs, suppressing the need of pre-enrichment step (Fig. 3).

Nucleic acid-based techniques can also be used for the detection and identification of CTCs. DNA or mRNA level detection of CTCs requires the designing of PCR based strategy using specific primers for individual tissue or organ, tumour transcripts or genetic mutations, translocations or methylation patterns exclusive to the tumour. Reverse transcription PCR (RT-PCR) based assays used for the detection of tumour-specific mRNA are one of the reference methods for detecting of low-abundance mRNA transcripts enabling sensitive quantification of CTC numbers (Fig. 3).

CTCs can also be detected with the help of various functional assays. The Epithelial ImmunoSPOT (EPISPOT) was introduced which enabled in vitro CTC detection [22] (Fig. 3). The presence of viable CTCs is assessed, using this technique, by growing them in short-term cell culture. The specific epithelial proteins which are secreted or released from these cells like PSA or cytokeratin 19 are detected by fluorescence. Quantitative information like numbers of viable CTCs as well as the qualitative information like presence or absence of CTC specific proteins may be gathered using this sensitive assay. The EPISPOT assay has been authenticated for several different cancers. Further improvement of this assay into a faster as well as more sensitive counterpart is being developed in a liquid microdroplet format, called EPIDROP, short for the EPISPOT in a drop, enabling the capture and detection of CTCs at the single-cell format [23]. In this method, after CTCs are stained they are individually encapsulated in fluid microdroplets. The total number of CTCs (EPCAM+ or EPCAM−), as well as the number of functional CTCs, are then estimated. Viable and apoptotic CTCs are distinguished by their EPCAM+ status. This also facilitates the portrayal of epithelial-mesenchymal transition (EMT). Strategies enabling subsequent molecular portrayal of the isolated CTCs are currently being incorporated into this inventive assay.

### Molecular Characterization of CTCs

Molecular characterization of CTCs might provide an improved understanding of key aspects of the metastatic process and may contribute to a personalized medicine approach. For in-depth analysis of CTCs, various innovative approaches have been developed in the past decade.

#### CTC Genomics

**Fig. 4:**
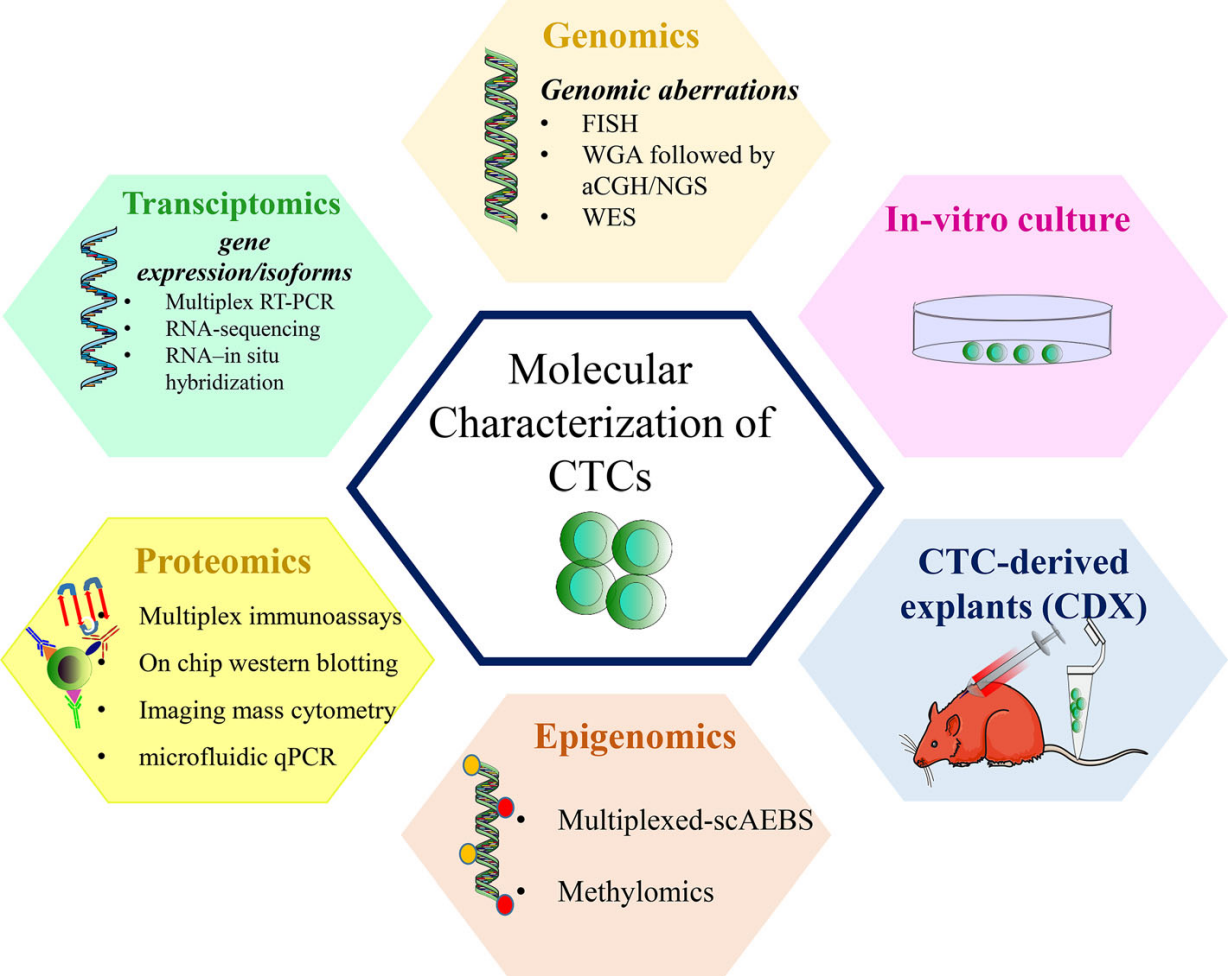
Molecular Characterization of CTCs. Various molecular features of CTCs can be characterized by using various recent technologies. CTC genomics can be studied by detecting various genomic aberrations using assays like FISH, WGA followed by aCGH/NGS and WES. CTC gene expression, as well as CTC-specific isoforms, can be demonstrated using technologies like multiplex RT-PCR, RNA-sequencing and RNA–in situ hybridization. The proteome of CTCs can be explored using recent advanced techniques such as multiplex immunoassays, on-chip western blotting, imaging mass cytometry and microfluidic qPCR. CTC-specific epigenomes can be studied using techniques like multiplexed-scAEBS and methylomics. Functional properties of CTCs such as their potential to form overt metastases could be explored by developing CDX models. In vitro, CTC cultures might be used for drug screening, personalized treatment and can provide insights into new pathways specific to metastasis-initiator CTCs and thus new therapeutic targets. FISH: Fluorescence in situ hybridizations; WGA: Whole genome amplification CGH; Array competitive genome hybridization; NGS: Next generation sequencing; WES: Whole exome sequencing; scAEBS: single-cell- agarose-embedded bisulfite sequencing; CDX- CTC-derived explants

The traditional approach for providing an insight to CTC genomics is to identify genomic aberrations. Genomic aberrations specific to CTCs can be identified by detecting these cells by immunostaining followed by fluorescence in situ hybridization (FISH). As CTCs are very rare as well as heterogeneous in nature; therefore more comprehensive characterization requires isolation of single-cell, followed by whole genome amplification to assess any copy number variations and detect specific mutations using array competitive genome hybridization or next-generation sequencing (NGS) technologies [24] (Fig. 4). However, watchful technical validation is required to circumvent false findings that are associated with DNA amplification protocol to ensure a low error rate. The findings of the single-cell analysis are challenging to be confirmed by alternative technique concurrently as single CTCs are limited resource, which is a natural drawback this strategy.

Notably, CTCs with EGFR aberrations were found in Non-Small Cell Lung Cancer (NSCLC) patients who had received EGFR tyrosine kinase inhibitor therapy [25]. Maheswaran et al reported 17 out of 18 CTC samples (94%) found to have mutations in the EGFR gene when analysed by The Scorpion Amplification Refractory Mutation System (SARMS) assay.

Androgen receptor plays a crucial role in the pathogenesis of castration-resistant prostate cancer (CRPC). Various genetic aberrations are identified in the CTCs isolated from these patients and many of these mutations were present in parent tumour itself, correlated with resistance to androgen-deprivation therapy [26]. These findings gave rise to the hypothesis that these mutations occurred even before the development of overt metastases. Therefore, the detection of these mutations in CTC genome could enable earlier intervention to avoid overt metastasis.

The SNaPshot assay is a combination of multiplex PCR amplification and multiplex primer extension which enable for the targeted detection of several mutations in one approach. CTC analysis from the patients receiving anti-HER2 therapy with lapatinib, a tyrosine kinase inhibitor, by this approach has identified the presence of PIK3CA (Phosphatidylinositol 4,5-bisphosphate 3-kinase catalytic subunit alpha isoform) mutations in 15.9% of the patients with metastatic breast cancer [27]. Mutations in PIK3CA may cause PI3K signalling pathway activation, resulting in resistance to HER2-targeted therapies in breast cancer patients [28].

Due to rarity and heterogeneity in CTCs, it is worth to study CTCs at the single-cell level. In prostate [29] as well as lung cancer [30], single-cell whole-exome sequencing explored the landscape of mutations in CTCs as a potential tool for CTC-based cancer diagnostics. As most of the Human DNA is not translated to mRNA but play important regulatory roles; thus, whole-exome sequencing has failed to provide a complete picture of the disease. This limitation can be overcome by analyzing whole genomes of the single CTCs. In this regard, Carter et al. sequenced whole genomes of single CTCs and showed that CTC copy number variation (CNV) profiles at relapse from five initially chemosensitive patients did not switch to a chemorefractory CNV status, indicating that the genetic basis of primary chemoresistance differs from acquired chemoresistance in small cell lung cancer [31]. CTC analysis from patients with localized primary tumours as well as those having distant metastasis showed mutations in genes which known to have potential driver role in development and progression of colorectal cancer [32]. Recently, Yu et al. [33] discovered a HER2 gene amplification/mutation in the single CTCs isolated from an advanced breast cancer patient by Whole-genome sequencing, which was not identified in the primary tumour. Therefore, recent studies suggest that genomic analysis of CTCs might help in making clinical decisions regarding personalized therapy and predicting the outcome.

#### CTC Transcriptomics

Usually, tissue-specific transcripts such as PSA are expressed by tumour cells (in prostate cancer), epithelial cytokeratins (in various epithelial cancers). After CTC enrichment, detection of these transcripts is not only specific tool for CTC detection, but also provide valuable information about phenotype of individual CTCs. These transcripts are usually detected by qRT-PCR assays and require cautious controls because contaminating leukocytes also have low-level extrinsic expression of the target transcripts, which might lead to false-positive results. Alternatively, the information on transcriptomics of CTCs can also be drawn using multiplex RT-PCR [34] or RNA-sequencing assays [34] (Fig. 4). These assays can also provide valuable insight on heterogeneity in individual CTCs isolated from the same patient [35, 36]. But the major challenge in studying transcriptomics is much less stability of RNA as compared to DNA. A validated protocol for reducing pre and post-analytical errors is required to be developed so as to avoid false-negative results arising from RNA degradation.

Evaluation of various Androgen Receptor (AR) splice variant by qRT-PCR of CTCs identified AR-V7 expression which is a constitutively active truncated Androgen Receptor (AR) splice variant deficient in the ligand-binding domain. Expression of this AR variant acts as a prediction marker for low response to anti-androgen therapy with abiraterone or enzalutamide [36, 37]. Contrary to this, chemotherapeutic drugs sensitivity (to taxanes) was unaffected by AR-V7 expression [38–40]. Taking background from these studies Scher et al. firstly provide evidence that CTCs can act as a predictive biomarker to decide between a taxane group of drugs versus an AR signalling inhibitor in the clinical setting [41, 42].

Adherent epithelial cells undergo a transition to a migratory mesenchymal state referred to as Epithelial-mesenchymal transition (EMT). This process of EMT has been associated with tumour metastasis in preclinical models. When EMT is characterized in circulating tumour cells isolated from breast cancer patients by RNA–in situ hybridization (ISH) assay, it was observed that sporadic primary tumour cells simultaneously expressed epithelial and mesenchymal markers, while the cells with mesenchymal phenotype were highly enriched in CTCs [43]. Presence of mesenchymal CTCs was associated with disease progression. These data support a role for EMT in the blood-borne metastasis of human breast cancer [43].

Again knowledge of CTC transcriptomics is incomplete without studying CTCs at a single-cell level as CTCs are very heterogeneous. Mouse and human pancreatic cancer-derived CTCs display elevated expression of extracellular matrix (ECM) proteins such as SPARC [44]. Knockdown of this SPARC protein in cancer cells suppresses their migration and invasiveness. The abnormal expression of stromal ECM genes in CTCs provides insight into their role in microenvironmental modulation facilitating the spread of cancer to distant organs [44]. In castration-resistant prostate cancer, analysis of CTCs through RNA sequencing identified additional mechanisms of AR inhibitor resistance [45]. In a recent study on small cell lung cancer, intratumoural heterogeneity was analysed by single-cell RNA sequencing of CTC-derived xenografts and patient CTCs identified their chemosensitive and chemoresistant phenotype [46]. It was observed that increased intratumoural heterogeneity, along with a heterogeneous expression of therapeutic targets as well as potential resistance pathways, are associated with treatment resistance. These findings suggest that single-cell RNA sequencing of CTCs can provide valuable information about various treatment resistance pathways and may prove to be significant prognostic as well as predictive biomarkers for therapy monitoring.

#### CTC Proteomics

Analysis of CTC proteome would complement transcriptomic and genomic characterization of these rare cells defining their phenotypic characteristics. Most widely used strategy for CTC characterization is immunophenotyping of CTCs using markers of proliferation/apoptosis but this is limited to only a limited number of proteins beyond those are required for enrichment and detection of the CTCs (Fig. 4).

Contrasting single-cell genomics and transcriptomics, developments in single-cell proteomics are minimalistic. Single-cell protein assays used currently are mostly single-stage traditional immunoassays which include enzyme-linked immunosorbent assays and immunocytochemistry, as well as more advanced immunoassay formats which have been developed for improving multiplexing using spatial barcode [47]. CTC protein analysis mainly focus on membrane-bound and secreted proteins rather than endogenous proteins [48]. Simultaneous estimation of multiple proteins in single-CTC is an important complement to single-CTC genomic and transcriptomic studies. A microfluidic western blot technology has been developed which enable the proteomic phenotyping of CTCs developed 8 detectable protein panel which includes GAPDH, β-tubulin, pan-CK, ERK, EpCAM, ER, eIF4E and low expression CD45. [49]. Recently, Gerdtsson et al. have developed HD-SCA (High Definition Single Cell Analysis) workflow with the consequent downstream multiplex proteomic analysis using imaging mass cytometry which allowed the multiplexing of around 40 proteins of CTCs at the single-cell level [50]. The major drawback of these technologies is that these are limited to the assessment of very less number of proteins. Thus, further advancement of cell-based proteomics strategies like proximity extension assay technology [51] might overcome this shortcoming. It is an immunoassay profile enabling detection of about 92 proteins in minute quantities of a biological sample. In this technique, two antibodies which are linked to oligonucleotides recognize each target protein. When these two antibodies bound to the same target molecules, a polymerase enzyme produces DNA reporter strands from two specific oligonucleotides, attached to the antibodies, enabling quantification of the products using microfluidic qPCR (Fluidigm^®^) which act as a measure of the amount of the target protein [51].

#### CTC Epigenomics

Epigenomics is an overview of the chemical modifications and conformations of DNA sequences within a cell, which are associated with epigenetic memory, cellular identity and tissue-specific roles. Majority of the current techniques in this field can provide epigenomic features across a large number of cells. But to understand the epigenetics within complex and heterogeneous cells like CTCs, development of single-cell epigenomics is a great need. Unlike CTC genomics as well as transcriptomics, the field of CTC epigenomics remains less explored. Analysing DNA methylation in the rare CTCs remains technically very challenging. Pixberg et al. [52] developed a single-cell AEBS (Agarose-embedded bisulfite sequencing) referred as scAEBS protocol combined with a PCR based strategy allowing multiplexed analysis of multiple loci (multiplexed-scAEBS) for analysing three EMT-associated genes miR-200c/141, miR-200b/a/429 and CDH1 in a single CTC. After this, various studies were performed that could provide comprehensive genome-wide DNA-methylation events, enabling characterization of CTCs. These studies provide important insights into the CTC biology and highlight an essential connection between DNA methylation dynamics and phenotypic features of CTCs (such as their ability to circulate as multicellular clusters) at critical stemness- and proliferation-related sites [53]. Studying epigenetic mechanisms at a single-cell resolution could thus provide new understandings into the basic biology of cancer metastases and has the potential to identify novel biomarkers for monitoring cancer progression, therapeutic response and might eventually result in epigenetic driven therapies. (Figure 4).

#### CTC-Derived Explants (CDXs)

CTCs can also be characterized functionally, by analysing their potential of homing secondary sites and ultimately forming overt metastases in mice models, called as CTC-derived explants (CDXs) models [54, 55]. The first CDX was derived from primary human luminal breast cancer CTCs and it allowed the identification of metastasis-initiating cells among CTCs [55]. Also, these CDX models can be used to test the susceptibility of various drugs that might act as valuable anticancer drugs [54]. For the first time Hogdkinson et al demonstrated that CTCs from patients with SCLC (Small Cell Lung Cancer) can form tumours in immunocompromised mice and these tumours have conserved morphological and genetic characteristics of parent CTCs. CDXs also shows similar responses to platinum and etoposide containing chemotherapeutic agents, what is seen in their donor patients. These unique mouse models offer unique systems for therapy testing and elucidating novel drug resistance mechanisms. The rate of the development of CDX models is minimal due to the prerequisite for a large number of CTCs e.g. more than 1000 CTCs from patients with breast cancer [55]. This limitation makes it difficult to use such models for personalized therapy. However, these models have shown to express similar molecular and cellular characteristics of the primary tumours and their response to chemotherapy [54, 55] (Fig. 4).

#### In-Vitro CTC culture

In-vitro culture of CTCs provides an alternate approach for functional characterization of CTCs. As CTCs are limited in number, therefore long term culturing of CTCs remains a challenge. Despite this, some groups have demonstrated that the establishment of permanent cell lines of CTCs isolated from patients with advanced disease is also a possibility. However, these cell lines have partially changed their phenotypes to those of parent tumour tissue but having maintained a specific molecular signature [56] which differ from parent tumour reflecting metastatic competency generally attributed to CTCs. Nevertheless, the CTC-derived cell lines have a high metabolic rate, stem cell-like characteristics, and a specific DNA repair phenotype [56]. These CTC cell line can potentially be used for drug screening, but there is a hurdle in using these cell line to draw a correct treatment decisions regarding treatment for the donor patients. Establishing these cell lines is not yet fast enough to use as a tool for personalized treatment. Additionally, a high CTC number is required to establish cell lines, limiting the use of these models. Indeed, the development of more advanced CTC isolation methods such as in vivo CTC capture devices [57], which enable higher CTC yields than any conventional enrichment strategy might overcome this limitation. Also, the short-term expansion of CTC cultures could provide pertinent information rapidly to guide treatment decisions for the patient. Such cultures might also provide insight into molecular pathways related to CTC initiated metastasis and thus new therapeutic targets (Fig. 4).

CTCs also get evolved with the advancement of disease as well as the progress of treatment. So, this poses another challenge for the development of cell lines that precisely replicate the real-time disease status. Thus the establishment of several CTC cell lines sequentially throughout disease process and treatment can provide new insight. In this regard, Soler et al. [58] demonstrated that sequential colon CTC lines established during treatment have similar traits, but CTC clones with discrete and isolated phenotypic characteristics are gained in due course of time.

### CTC as a Snapshot of Tumour Heterogeneity

In principle, there are two types of tumour heterogeneity, temporal and spatial tumour heterogeneity and both are the outcome of tumour progression. When tumour progresses from localized to metastatic subtype is referred to as temporal tumour heterogeneity and this is characterized by the heterogeneity amongst the primary tumour and metastatic lesions. However, spatial tumour heterogeneity is the outcome of the presence of different tumour clones within the primary tumour of or between metastatic lesions arising in different organs of the same patient. Understanding of this complex biology of tumour heterogeneity requires detailed sampling of every metastatic lesion by multiple and repeated biopsies and an in-depth analysis of the primary and metastatic tumour. The feasibility of this practice is always questionable as biopsies are often limited to a small number of sampling time points and accessible sites. A liquid biopsy might be more useful to understand tumour heterogeneity. CTCs represent the entire spectrum of mutations present in the primary tumour and distal metastases [59]. It suggests the utility of CTCs for being used as a biomarker to reveal a certain type of cell populations sensitive to various therapies. However, molecular characterization of CTCs was initially performed on enriched fractions of peripheral blood [60] which failed to provide significant information on tumour heterogeneity. It is also more difficult to identify rare clones in CTC enriched fractions. In this regard, the study of CTCs at single-cell resolution is, therefore, the method of choice to act as a marker for tumour heterogeneity.

With the current advances in single-cell analysis strategies, studies of CTCs at single- cell level may provide a minimally invasive approach to depict and monitor dynamic variations in tumour heterogeneity at the genome, transcriptome, proteome and functional levels. Recently, a sophisticated process has been developed to isolate and sequence the whole exomes of CTCs at single-cell resolution with high accuracy. This allowed the comparison of single CTCs and multiple regions of the primary tumour in patients [29]. The mutation profile identified in CTCs and overt metastases closely resembled each other as well as with the one particular area of the primary tumour, suggesting this area of primary to be the potential metastasis-initiating area [29]. Single-cell RNA-sequencing (RNA-Seq) profiles of CTCs in prostate cancer illustrated the CTC heterogeneity in various signalling pathways that may have contributed to the treatment resistance [61]. In some cases, it was demonstrated that it is simpler to characterize tumour heterogeneity from CTCs rather than primary tumours. For example, diagnostic leukapheresis (DLA), has enabled the analysis of hundreds of CTCs simultaneously which helped to identify an obvious tumour heterogeneity including subclonal copy-number alterations (CNAs) which were not easily identified from the analysis by regular tumour biopsies [62]. In metastatic breast cancer, individual and pooled CTCs were sequenced for a 130 gene panel and compared with their metastatic counterparts. Nearly 85% of similarity was observed in at least one or more somatic mutations and copy number variations [63]. In general, these data demonstrated that sequencing of CTCs could provide significant pertinent information on tumour heterogeneity and metastatic development, which might reveal strategies that could be used to prevent or treat metastasis. Therefore, the depiction of CTCs at single- cell level could provide mechanistic understandings into the heterogeneity of metastasis.

### The Emerging Trend in CTC-Directed Diagnosis

#### CTCs in the Prediction of Micro-metastasis

Within 5 years of primary tumour removal, a considerable fraction of cancer patients have relapse of the disease in spite of initially being free of noticeable metastasis. Late relapses can occur in breast cancers with hormone receptor-positivity, that is regarded as the prototype cancer associated with late relapses. The 20-year risk of distant reappearance of cancer is 13% in those with no nodal involvement (T1N0) among the patients with stage T1 disease, 20% in those with 1–3 involved nodes (T1N1–3) and 34% in those with 4–9 involved nodes (T1N4–9) and the patients with stage T2 disease, the risks of distant recurrence are 19%, 26% and 41%, respectively [64]. Thus, a substantial number of patients who have gone through successful removal of primary early-stage cancer may already have occult metastases at the microscopic level or otherwise termed as a minimal residual disease (MRD) that continues to be there even after the therapy as a possible source of consequent relapse. Recent findings suggest that the finding of CTCs in patient blood even after years of initial diagnosis and successful treatment might indicate relapse earlier than standard clinical procedures.

The detection of CTCs at nearly 5 years after initial diagnosis can predict late recurrence in patients having operable hormone receptor-negative breast cancer [65]. The discovery of CTCs is associated with a 13.1-fold higher risk of relapse as illustrated by multivariate analysis [65]. In 193 patients, however, only 1 patient who had no detectable CTCs but developed disease recurrence [65].

In non-metastatic colorectal cancer, the occurrence of CTCs weeks after surgery and before the start of adjuvant therapy was not associated to clinical outcome, whereas detection of CTCs 2–3 years after surgery were able to successfully predict an unfavourable prognosis [66]. This indicated that the long-term persistence of minimal residual disease is reflected by the finding of CTCs.

A prospective study on 243 patients with melanoma has demonstrated that the finding of one or more CTCs per 7.5-mL of blood at baseline can independently predict the relapse within 6 months of presentation, as well as at 54-month follow-up [67]. This study provided evidence that CTC detection at first clinical presentation is useful to recognize patients with stage III melanoma who might be benefited from extensive imaging surveillance, or adjuvant systemic therapy [67]. Kantara et al. have developed a novel diagnostic assay for detecting cancer stem cell fraction of CTCs in circulation. Detection of circulating cancer stem cells was more accurately predicted the risk of relapse or metastatic disease in a patient [68]. Approximately 50% of patients with resectable pancreatic cancer had circulating tumour cells during time of surgery, mainly in portal vein and detection of CTCs in the portal vein predicted liver metastases [69].

But studies which utilized molecular characteristics of CTCs to predict relapse or metastases are lagging. In a recent case report on exome sequencing of pulmonary venous CTCs (PV-CTCs) isolated from non-small cell lung cancer (NSCLC), during the surgery showed that CTCs are related with the subsequent metastatic disease rather than primary tumour [70]. This highlights the novel role of PV-CTCs for early prediction of NSCLC recurrence after surgery [70].

Recent studies suggested that assessment of CTCs might provide valuable information about the patients at high risk of relapse, as well as suggest predictive information regarding potential therapies and their effectiveness to significantly reduce the possibility of relapse and improve clinical outcome.

#### CTCs as Prognostic Marker

The enumeration of CTCs has proved to be an operative means for prediction of disease aggressiveness and monitoring of therapeutic response with a minimally invasive procedure [71]. CTCs detected with Cell Search technology have been clinically validated in prognosticating various cancers like breast cancer [72], colorectal cancer (CRC) [73], prostate cancer [74], small cell lung cancer (SCLC) [75], non-small cell lung cancer (NSCLC) [76] and pancreatic adenocarcinoma [77]. The prognostic role of CTCs enumeration is also being evaluated in many other cancer types including oesophageal squamous cell carcinoma [78], skin Cancer (Merkel cell) [79], advanced urothelial carcinoma [80].

On the other hand, the establishment of the molecular signatures of the CTCs may contribute to an improved understanding of key aspects of the metastatic process and might act as a valuable prognostic tool to foster personalized medicine approaches. Recent studies have explored the genome, transcriptome as well as proteome based molecular features of CTCs to provide insight into their prognostic importance. Functional properties like cluster formation in short term culture of CTCs isolated from lung, breast, oesophagal and bladder cancer patients was shown to be linked with disease progression and overall reduced survival [81]. Recently, the role of CTC transcriptomics as predictive as well as a prognostic marker has been explored in various cancers. PD-L1 overexpression in CTCs has shown to be an important prognostic marker for the patients treated with the immune checkpoint inhibitor like nivolumab in breast cancer [82], head and neck squamous cell carcinoma [83] or non small cell lung cancer [84]. CTCs expressing high levels of proliferation markers are associated with poor progression free as well as overall survival and also may be used to monitor disease progression [85].

The expression of EGFR receptor in CTCs has been shown to be a predictive marker for non small cell lung cancer prognosis who are undergoing second-line therapy with AXD929 (EGFR tyrosine kinase inhibitor) [86].

#### CTCs in Cancer Staging

The use of CTCs in breast cancer staging has been included in the 4th edition of the WHO Classification of Tumours of the Breast [87] and in the 8th edition of the American Joint Committee on Cancer (AJCC) cancer staging manual [88]. Stage cM0(i+) has been included between stage M0 and M1 for the cases in which no clinical or radiographic evidence of distant metastases, but deposits of molecularly or microscopically detected tumour cells in circulating blood, bone marrow or other non-regional nodal tissue that are no larger than 0.2 mm in a patient without symptoms or signs of metastases. Moreover, Cristofanilli et al. [89] have proposed to further include a sub-classification for metastatic breast cancer stage IV into “indolent” and “aggressive” type according to the cut-off of ≥ 5 CTCs per 7.5 mL of blood after analysis of 18 cohorts of patients. They also suggested that the enumeration of CTCs is independent of clinical and molecular variants [89]. Again it was demonstrated that the information on the presence of CTC may provide new and independent prognosis staging information in ovarian cancer also [90].

## Conclusions and Future Perspectives

Analysis of CTCs is an easily repeatable, minimally-invasive and cost-effective approach for prediction as well as a real-time assessment of disease progression and monitoring cancer therapy. This liquid biopsy-based approach might overcome the intrinsic limitations of obtaining repeated biopsies from the primary tumour and metastatic tissue. Detection and enumeration of CTCs have proven to be a valuable tool for prognosis and prediction of cancer progression. But the characterization of molecular features of CTCSs could lead to a better understanding of critical aspects of the metastatic process and might provide additional information about disease progression that is not provided by CTC enumeration. For in-depth characterization of CTCs, various innovative approaches have been developed in the past decade which allowed the genetic/epigenetic/molecular/functional profiling of CTCs even at single cell resolution. Due to the continuous alteration in the genetic and molecular characteristics of cancer cells in the tumour microenvironment, the single-cell analysis of CTCs is an important aspect to elucidate the tumour heterogeneity in a real-time scenario and might allow adapting novel therapeutic approach during the treatment. For an overall better understanding of tumour heterogeneity, future studies are needed focusing on the amalgamation of different multi-omic approach on the same cell, as described by Han et al. and S. Bian et al. [91, 92]. They used a single-cell triple omics sequencing technique called scTrio-seq which illustrated the complex contribution of genomic as well as epigenomic heterogeneities in developing heterogenous transcriptomic character within a single cell. Studying molecular features of CTCs could help in designing better diagnostic (Cancer staging), prognostic as well as predictive tools for management of the disease. Thus, analysis of CTCs as a component of liquid biopsy have the potential to change the diagnostic, prognostic and predictive landscape in oncology.
